# Women’s Reasons to Seek Bariatric Surgery and Their Expectations on the Surgery Outcome — a Multicenter Study from Five European Countries

**DOI:** 10.1007/s11695-022-06280-w

**Published:** 2022-09-23

**Authors:** Mari Hult, Wouter te Riele, Lars Fischer, Signe Röstad, Kai Orava, Timo Heikkinen, Rune Sandbu, Anne Juuti, Stephanie E. Bonn

**Affiliations:** 1 Department for Upper GI Diseases, Karolinska University Hospital, Huddinge, Sweden; 2 Unit of Gastroenterology, Department of Medicine (Huddinge), Karolinska Institutet, Stockholm, Sweden; 3 Department of Surgery, Sint Antonius Hospital, Nieuwegein, The Netherlands; 4 Department of General, Visceral- and Metabolic Surgery, Baden-Baden, Germany; 5 Department of Surgery, Vestfold Hospital Trust, Vestfold, Norway; 6 Department of Surgery, Seinäjoki Central Hospital, Seinäjoki, Finland; 7 Department of Surgery, Suomen Terveystalo Oy, Oulu, Finland; 8 Abdominal Center, Helsinki University Hospital, Helsinki, Finland; 9 Division of Clinical Epidemiology, Department of Medicine (Solna), Karolinska Institutet, T2, Karolinska University Hospital, 171 76 Stockholm, Sweden

**Keywords:** Obesity, Bariatric surgery, Expectations, Weight loss

## Abstract

**Purpose:**

Understanding patients’ reasons for having bariatric surgery and their expectation on surgery outcomes is important to provide the best clinical practice and reduce unrealistic expectations. It is unknown if reasons and expectations differ between countries. We aimed to investigate the reasons for seeking bariatric surgery and expectations of surgical outcomes among patients in five European countries.

**Methods:**

In total, 250 women accepted for bariatric surgery were recruited: 50 women each from Finland, Germany, Norway, Sweden, and the Netherlands. Participants ranked 14 reasons for seeking surgery, and reported the three primary reasons. They also reported expectations on weight loss and impact of surgery vs. lifestyle on weight loss outcomes.

**Results:**

Mean age and body mass index were 42.9 ± 11.5 years and 45.1 ± 6.2 kg/m^2^, respectively. Weight loss and improved co-morbidity were ranked as the most important reasons. Participants expected to lose between 70.8 and 94.3% of their excessive weight. The expected impact of surgery as a driver of weight loss was higher in Germany and the Netherlands compared to in Finland, Norway, and Sweden where participants expected lifestyle changes to also have an impact.

**Conclusion:**

Weight loss and improved co-morbidities were the main reasons for undergoing bariatric surgery. Expectations on weight loss were generally very high, but expectations of surgery vs. lifestyle as the main driver of weight loss differed between countries. While some patients understand the importance of lifestyle change and maintenance of a healthy lifestyle after surgery in order to obtain a successful weight loss, other may need additional counselling.

**Graphical Abstract:**

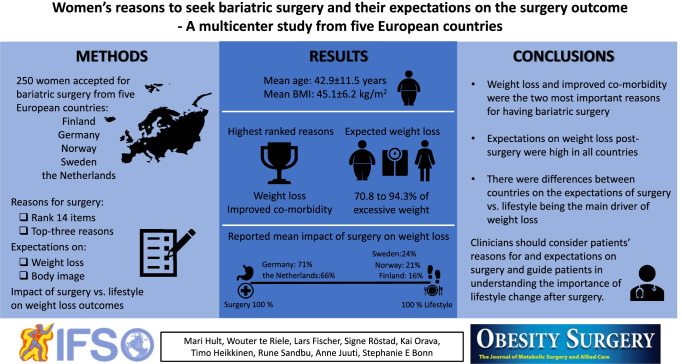

**Supplementary Information:**

The online version contains supplementary material available at 10.1007/s11695-022-06280-w.

## Introduction

Bariatric surgery improves morbidities such as diabetes and cardiovascular disease, and lowers the risk of developing cancer [1, 2]. It is associated with a low risk of complications [3–5], and has been proven more effective than conservative treatment for long-term weight loss and control of type 2 diabetes [6, 7].

Patients’ expectations of weight loss after bariatric surgery are often unrealistically high [8–13] and weight regain is common [14, 15]. What constitutes a realistic or unrealistic weight loss is often defined using the Goals and Relative Weights questionnaire [16]. Patients have been seen to report “dream weights” corresponding to 89% [9, 12], or even 99% [11], of excess weight loss. Given that a realistic expectation has been estimated to be 50–80%, depending on type of surgery [10], expectations are high. There is a tendency among women to have higher, and often more unrealistic, expectations on weight loss than men [9, 10, 17].

In conventional treatment of obesity, higher expected weight loss has been associated with improved weight loss [18]. Similar results have been shown during the first year after bariatric surgery [13, 19]. Nevertheless, weight loss expectations pre-surgery does not seem to predict weight loss post-surgery [8], but may affect other types of outcomes. For example, a prospective study following up on items of satisfaction 1 year after bariatric surgery showed that while weight loss was the most reported reason to seek surgery, the most reported item of satisfaction was improved self-esteem [20]. Pre-operative expectations on psychosocial outcomes, for example physical functioning and social appearance, were, however, not related to weight loss in patients after laparoscopic adjustable gastric banding [21].

To provide the best clinical practice and reduce the risk of unrealistic expectation on bariatric surgery outcomes, it is important to understand patients’ reasons for having surgery as well as their expectation on outcomes. Improved co-morbidities and physical health are often reported as important reasons for choosing to undergo bariatric surgery, both in quantitative [11, 12, 22, 23] and qualitative [24, 25] research. It is also common that patients expect the resulting weight loss to impact psychosocial factors, such as personal identity and relationships [25]. While some have shown women to expect more positive effects on both social and physical attributes than men [9], others have found reasons and expectations to be similar between the sexes [26]. Studies on reasons and expectations of patients in different countries are available, but direct comparisons between countries have, to the best of our knowledge, not been made. Potential differences between countries may exist due to external factors, such as culture, or different referral pathways and pre-operative information. To better understand the international literature in order to best tailor information directed at patients planning to undergo bariatric surgery, we must be aware if such differences exist.

In this international multicenter study, we aimed to investigate the reasons for seeking bariatric surgery and patients’ expectations of surgical outcomes among women in Finland, Germany, Norway, Sweden, and the Netherlands.

## Methods

This study was a cross-sectional multicenter study carried out in Finland, Germany, Norway, Sweden, and the Netherlands. In total, 250 women were included. In all countries except Germany, 50 women accepted for bariatric surgery between January 2012 and January 2013 were recruited. In Germany, a larger study comprising also men was conducted [26]. To make data between the countries comparable, 50 female participants included in the German study were included in this study. There was no systematic recording of number of patients invited or number of patients that declined participation in any of the countries.

Criteria for inclusion were acceptance to surgery according to the international guidelines [27], i.e., a body mass index (BMI) > 40 kg/m^2^ or a BMI > 35 kg/m^2^, and a co-morbidity. Patients were excluded if they previously had bariatric surgery or balloon, were < 18 years of age, or were unable to read or understand the language of the study questionnaire (i.e., Finnish, German, Norwegian, Swedish, or Dutch).

A questionnaire designed specifically for this study was distributed to the patients some weeks before surgery. Details about the development of the questionnaire and the included questions have been published elsewhere [26]. In brief, the questionnaire was developed in a collaboration between bariatric surgeons, endocrinologists, and statisticians from the five included countries. It was originally developed in English and thereafter translated and validated in each of the five languages according to protocol [28]. The comprehensibility and ambiguity of the questionnaire was tested in a pilot study (*n* = 10 in each country) and no comments indicating that the questionnaire was incomprehensible were made [26].

In the questionnaire, participants were asked to self-report age, height, weight, civil status, children, smoking, current occupation, level of education, co-morbidities, and medication use. BMI (kg/m^2^) was calculated based on reported height and weight.

### Reasons for Seeking Bariatric Surgery

Participants were asked to rank 14 different reasons to seek surgery from 1 (not important) to 5 (very important) on 5-step Likert scale. The 14 reasons listed were presented in different orderings to the participants in order to prevent systematic errors. Participants were thereafter asked to, from the 14 listed reasons, name the most important, the second most important, and the third most important reason to seek surgery.

In order to identify the most important reasons for seeking surgery, a total score was calculated for each of the 14 reasons based on participants’ listings of the top three reasons. In calculations, a reason listed as the most important was given ten points, a reason listed as the second most important five points, and a reason listed as the third most important scored three points in order to create a summary score where more weight was given to the reason ranked as most important. The reasons not reported as top three by a participant were given 0 points in the scoring. The sum of all participants scoring was thereafter calculated.

### Expectation on Surgery Outcome

Participants’ excessive weight was calculated as the number of kilograms above the weight corresponding to a BMI of 25 kg/m^2^ based on the self-reported weight in the questionnaire. Participants were also asked to report their expected weight loss after surgery in kilograms by responding to an open question asking “How many kilograms do you expect to lose after surgery.” This was then used to calculate the expected percentage of excessive weight loss (%EWL) according to the formula: expected weight loss/excessive weight. To evaluate the influence of pre-surgery weight on expectations, comparisons of percent expected weight loss were made between categories of BMI < 45 kg/m^2^ and ≥ 45 kg/m^2^.

Participants were asked to identify their current and their expected body shape post-surgery using Stunkard silhouettes, i.e., standardized drawings of different body shapes [29, 30]. They were also asked to report the effect they expected the surgery to have, contrary to how much of the effect would be due to lifestyle changes after surgery. Participants reported this by making a mark on a 10 cm long scale ranging from 0 (0% of the expected effects due to surgery) to 10 (100% of the expected effect due to surgery).

### Statistical Analysis

Characteristics of study participants by country are presented as mean and standard deviation (SD), or number and percentage for continuous and categorical variables, respectively. Results of scoring of the top three reasons to seek surgery, Stunkard silhouettes, and variables of reported excessive weight and expected weight loss are shown as mean (SD). Differences in scoring of reasons between countries and differences in expected percentage of excessive weight loss by BMI categories (< 45 vs. ≥ 45 kg/m^2^) were analyzed using the Kruskal–Wallis test. Descriptive statistics showing number and percentage of participants in different categories of expected impact of surgery on weight loss outcomes by country were assessed. Statistical significance was defined as *p*-values < 0.05. All analyses were performed using STATA 14.2 (StataCorp LP, College Station, Texas).

## Results

Characteristics of study participant are presented in Table 1. The mean age of all study participants was 42.9 ± 11.5 years, with Finland having the highest (48.0 ± 9.5 years) and Sweden the lowest (37.5 ± 10.4) years. The mean BMI of all participants was 45.1 ± 6.2 kg/m^2^, with Germany having the highest (48.6 kg/m^2^) and Sweden the lowest (40.8 kg/m^2^). The majority of participants (≥ 60% in all countries) were married/had a partner, and had children. Most participants were employed; > 50% in all countries except Norway where fewer (44%) were employed. On average, 16% had a university degree, with the highest proportion in Sweden (32%), and the lowest in the Netherlands (6%). Medical treatment for diabetes or hypertension were common among participants in all countries except in Sweden where this was less prevalent.

**Table 1:** Characteristics of all study participants and by country

	Finland (*n* = 50)		Germany (*n* = 50)		Norway (*n* = 50)		Sweden (*n* = 50)		The Netherlands (*n* = 50)	
	Mean	(SD)	Mean	(SD)	Mean	(SD)	Mean	(SD)	Mean	(SD)
Age (years)	48.0	(9.5)	41.1	(11.0)	46.5	(9.9)	37.5	(10.4)	41.5	(13.3)
BMI (kg/m^2^)	45.0	(6.5)	48.6	(6.4)	45.6	(6.2)	40.8	(5.3)	45.6	(3.9)
	*n*	(%)	*n*	(%)	*n*	(%)	*n*	(%)	*n*	(%)
Civil status^1^										
Single	17	(33.0)	17	(34.0)	15	(30.0)	19	(38.0)	7	(14.0)
Married/partner	28	(56.0)	33	(66.0)	35	(70.0)	30	(60.0)	43	(86.0)
Children^1^										
Yes	32	(64.0)	32	(64.0)	31	(62.0)	35	(70.0)	39	(78.0)
No	14	(28.0)	18	(36.0)	10	(20.0)	12	(24.0)	11	(22.0)
Smoking^1^										
Yes	11	(22.0)	11	(22.0)	5	(10.0)	7	(14.0)	9	(18.0)
No	33	(66.0)	37	(74.0)	36	(72.0)	40	(80.0)	41	(82.0)
Occupation^1^										
Working	33	(66.0)	29	(58.0)	22	(44.0)	33	(66.0)	28	(56.0)
On sick-leave	4	(8.0)	3	(6.0)	14	(28.0)	2	(4.0)	4	(8.0)
Retired	6	(12.0)	4	(8.0)	2	(4.0)	0	-	1	(2.0)
Unemployed	1	(2.0)	4	(8.0)	8	(16.0)	5	(10.0)	10	(20.0)
Education^1^										
No university degree	37	(74.0)	44	(88.0)	35	(70.0)	25	(50.0)	30	(60.0)
University degree	8	(16.0)	5	(10.0)	9	(18.0)	16	(32.0)	3	(6.0)
Medication/treatment for										
Diabetes — any	24	(48.0)	19	(38.0)	20	(40.0)	2	(4.0)	12	(24.0)
Diabetes — insulin	11	(22.0)	8	(16.0)	11	(22.0)	1	(2.0)	7	(14.0)
High blood pressure	29	(58.0)	25	(50.0)	20	(40.0)	9	(18.0)	21	(42.0)
High blood lipids	14	(28.0)	4	(8.0)	16	(32.0)	7	(14.0)	7	(14.0)
Sleep apnea with CPAP-mask	6	(12.0)	7	(14.0)	19	(38.0)	0	-	3	(6.0)
Depression	6	(12.0)	15	(30.0)	11	(22.0)	16	(32.0)	4	(8.0)
Joint pain^2^	19	(38.0)	20	(40.0)	18	(36.0)	12	(24.0)	18	(36.0)
Infertility or PCOS	5	(10.0)	4	(8.0)	6	(12.0)	6	(12.0)	7	(14.0)

^1^Missing data: civil status *n* = 6; children *n* = 16; smoking *n* = 20; occupation *n* = 37; education *n* = 38

^2^Pain related to arthrosis or other musculoskeletal disorder

*BMI*, body mass index; *CPAP*, continuous positive airway pressure; *PCOS*, polycystic ovarian syndrome

### Reasons to Seek Bariatric Surgery

Almost all participants ranked weight loss (233/250, 93.2%), increased life expectancy (231/250, 92.4%), and improved physical activity (216/250, 86.4%) as very important (4 or 5 on the Likert scale). Most of the remaining reasons were also ranked as very important by a majority of participants: improved mental health (189/250, 75.6%), improved co-morbidities (182/250, 72.8%), improved self-esteem (179/250, 71.6%), pain reduction (176/250, 70.4%), reduction in clothing size (175/250, 70.0%), better work performance (156/250, 62.4%), improved intimacy (153/250, 61.2%), reduced need for medication (151/250, 60.4%), improved social life (141/250, 56.4%), increased chance of employment (100/250, 40.0%), and improved fertility (49/250, 19.6%). Corresponding numbers of participants rating reasons for surgery as very important using only the highest rank (i.e., 5 on the Likert scale), among all participants and per country, are shown in Supplementary table 1. Weight loss was rated as very important by the most participants in all countries.

Results from the scoring of the participants reported top three reasons for seeking bariatric surgery are shown in Table 2. Weight loss and improved co-morbidity had the highest mean scores among all participants. Weight loss had the highest score in Norway, Sweden, and the Netherlands, where improved co-morbidities were ranked second. Improved co-morbidities had the highest scores in Finland and Germany, but while weight loss was ranked as the second most important reason in Finland, pain reduction was ranked second in Germany. Even if weight loss had a high score in all countries, it was statistically significantly different between countries (*p* = 0.0001). For example, in the countries where weight loss had the highest score, it was 3.12 ± 4.0 and 4.18 ± 4.3, Sweden and Norway, respectively, compared to 6.56 ± 4.0 in the Netherlands. More than half of participants in the Netherlands (27/50, 54%) reported weight loss as the most important reason. Corresponding proportions ranking weight loss first ranged between 18 and 34% in the other countries. There score for improved co-morbidities did not differ between countries (*p* = 0.45), and the proportion of participants ranking it first varied between 18 and 30% in all countries. Improved life expectancy, reduced pain, improved self-esteem, and improved physical activity had high scores in all countries, although the mean score for improved physical activity differed significantly (*p* = 0.0004) with no participants in the Netherlands ranking it as the most important reason. Although in general not ranked as a top reason, there were significant differences (*p* < 0.05) between the countries in the scores of improved mental health, reduced need for medication, improved work performance, increased chance of employment, and reduction in clothing size.

**Table 2:** Results of the scoring of top three reasons to seek surgery among all participants and by country, listed by ranking of importance by all participants (highest rank on top)

Item to improve	All		Finland		Germany		Norway		Sweden		The Netherlands		*p*^1^
	Mean	(SD)	Mean	(SD)	Mean	(SD)	Mean	(SD)	Mean	(SD)	Mean	(SD)	
Weight loss	4.0	(4.3)	3.4	(4.2)	2.5	(3.9)	4.2	(4.3)	3.1	(4.0)	6.6	(4.0)	< 0.001
Improved co-morbidity^2^	3.2	(4.2)	3.6	(4.5)	3.3	(4.2)	2.4	(3.9)	2.9	(4.1)	3.9	(4.4)	0.45
Increased life expectancy	2.0	(3.2)	2.7	(3.7)	1.1	(2.1)	1.8	(3.2)	1.9	(3.2)	2.3	(3.3)	0.13
Pain reduction	1.7	(3.2)	1.1	(2.4)	2.8	(3.9)	1.7	(3.2)	1.7	(3.4)	1.4	(2.5)	0.11
Improved self esteem	1.3	(2.7)	1.2	(2.9)	1.4	(2.7)	1.2	(2.2)	1.8	(3.4)	1.0	(1.8)	0.80
Improved physical activity	1.2	(2.7)	2.0	(3.0)	1.8	(3.4)	0.9	(2.3)	1.3	(2.6)	0.1	(0.6)	< 0.001
Improved social life	0.7	(1.9)	0.3	(1.1)	1.0	(2.4)	0.8	(2.3)	0.7	(1.8)	0.6	(1.6)	0.64
Improved mental health	0.7	(2.1)	0.4	(1.1)	1.3	(2.9)	0.1	(0.4)	1.6	(3.1)	-	-	< 0.001
Reduced need for medication	0.6	(1.7)	1.0	(2.2)	0.5	(1.5)	0.9	(1.9)	-	-	0.6	(1.7)	0.02
Better work performance	0.5	(1.7)	1.0	(2.2)	0.5	(1.4)	0.9	(2.6)	0.1	(0.4)	0.1	(0.6)	0.008
Improved fertility	0.4	(1.5)	0.4	(1.6)	0.4	(2.0)	0.1	(0.6)	0.5	(1.8)	0.4	(1.1)	0.51
Increased chance of employment	0.3	(1.1)	-	-	0.2	(0.9)	0.8	(1.9)	0.3	(1.1)	0.2	(0.9)	0.02
Improved intimacy and partnership	0.2	(1.2)	0.18	(0.7)	0.5	(1.7)	0.1	(0.6)	0.5	(1.7)	-	-	0.32
Reduction in clothing size	0.2	(0.8)	0.18	(0.7)	0.3	(1.1)	0.1	(0.4)	0.5	(1.2)	-	-	0.03
Other reason	0.2	(1.0)	0.20	(1.4)	-	-	-	-	0.2	(0.9)	0.4	(1.4)	0.08

^1^*p*-value from Kruskal–Wallis test comparing all countries

^2^Such as diabetes, hypertension, high blood lipids, and sleep apnea

Results of excessive weight (kg), expected weight loss (kg), and expected percentage of excess weight loss (%EWL) are shown in Table 3. Overall, participants had a mean excessive weight of 56.2 ± 17.9 kg and expected to lose an average of 42.4 ± 12.9 kg after surgery, corresponding to an expected %EWL of 79.2. There was a statistically significant difference in expected %EWL between the countries (*p* = 0.0001), with Swedish participants expecting to lose the most (%EWL: 94.3), and Finnish and German participants expecting to lose the least (%EWL: 70.8 and 71.7, respectively). Participants with a pre-surgery BMI < 45 kg/m^2^ expected a significantly higher %EWL compared to participants with a BMI ≥ 45 kg/m^2^ (%EWL: 86.8 vs. 70.3, *p* = 0.0001). This pattern was seen in all countries except among participants in Germany where no difference in expected %EWL between BMI categories (72.4 vs. 71.3, *p* = 0.91).

**Table 3:** Excessive weight and reported expected weight loss post-surgery

	Finland		Germany		Norway		Sweden		The Netherlands	
	Mean	(SD)	Mean	(SD)	Mean	(SD)	Mean	(SD)	Mean	(SD)
All	(*n* = 49)		(*n* = 50)		(*n* = 49)		(*n* = 50)		(*n* = 49)	
Excessive weight, kg	54.8	(17.9)	64.9	(17.0)	57.8	(17.8)	43.8	(16.1)	59.5	(13.7)
Expected weight loss, kg	36.7	(10.3)	44.8	(13.6)	46.3	(11.3)	40.7	(14.4)	43.7	(12.6)
Expected %EWL	70.8	(22.2)	71.7	(19.5)	83.0	(17.5)	94.3	(19.5)	75.4	(18.1)
BMI < 45 kg/m^2^	(*n* = 26)		(*n* = 17)		(*n* = 26)		(*n* = 41)		(*n* = 23)	
Excessive weight, kg	40.9	(6.5)	49.9	(6.6)	43.6	(8.2)	37.8	(8.8)	51.0	(10.9)
Expected weight loss, kg	32.4	(7.4)	36.1	(10.7)	41.3	(8.1)	35.9	(9.7)	41.1	(12.1)
Expected %EWL	80.3	(21.9)	72.4	(19.5)	93.3	(13.4)	96.0	(20.6)	81.2	(14.3)
BMI ≥ 45 kg/m^2^	(*n* = 23)		(*n* = 33)		(*n* = 23)		(*n* = 9)		(*n* = 26)	
Excessive weight, kg	70.5	(12.7)	72.6	(15.4)	73.9	(10.4)	71.5	(12.3)	66.9	(11.5)
Expected weight loss, kg	41.7	(11.1)	49.8	(12.6)	52.7	(10.8)	61.9	(12.5)	46.5	(12.7)
Expected %EWL	60.1	(17.3)	71.3	(19.9)	71.9	(14.4)	87.0	(11.7)	70.6	(19.7)
Difference in expected % excessive weight loss between BMI < 45 kg/m^2^ and ≥ 45 kg/m^2^ *p*-value from Kruskal–Wallis test										
	0.002		0.91		< 0.001		0.07		0.08	

*EWL*, expected weight loss

### Expectations on Surgery Outcome

**Fig. 1:**
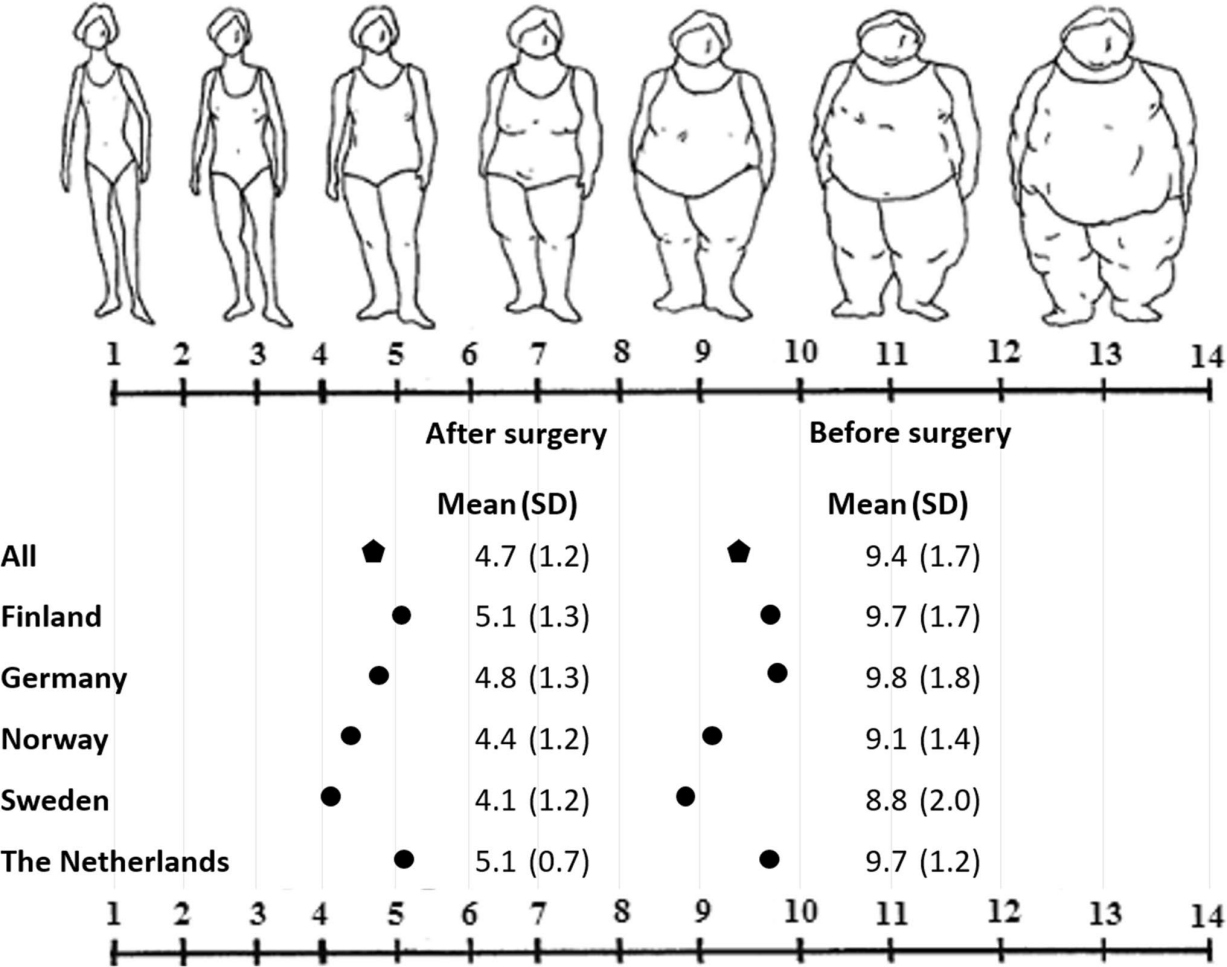
Patients self-reported perceived body image using the Stunkard silhouettes before surgery and their expected perceived ideal body image after surgery. The average scores with standard deviation (SD) for all participants (*n* = 234) and by country are shown. Figures are reproduced with permission

Results from participants self-reported perceived body image using the Stunkard silhouettes before surgery and their expected perceived ideal body image after surgery are presented in Fig. 1. The average reported figure on the scale of silhouettes was 9.4 ± 1.7 pre-surgery, and the expected silhouette was 4.7 ± 1.2, among all participants. Pre-surgery silhouettes corresponded with current BMI. For example, participants in Germany had the highest pre-surgery BMI (48.6 kg/m^2^) and scored highest on the silhouette scale (9.8 ± 1.7), while participants in Sweden that had the lowest pre-surgery BMI (40.8 kg/m^2^) also reported the smallest silhouette (8.8 ± 2.0). The mean differences in pre-surgery and expected post-surgery silhouettes ranged between 4.6 ± 1.3 (the Netherlands) and 5.0 ± 1.8 (Germany) in the different countries. There was no statically significant difference in expected difference between countries (*p* = 0.85).

Expectations on how much of the expected weight loss after surgery that would be due to surgery itself vs. lifestyle changes differed between countries. In Germany and the Netherlands, a majority of participants expected surgery to have the greatest impact and 35/49 (71.4%) and 31/47 (66.0%) participants, respectively, reported that 80% or more of the expected weight loss would be due to surgery. The corresponding numbers were substantially lower in Finland (7/44, 15.9%), Norway (10/47, 21.3%), and Sweden (12/50, 24.0%). Very few participants in Germany, 1/49 (2.0%), and the Netherlands, 5/47 (10.6%), reported an impact of surgery below 20%, while this was more often the case in Finland (12/44, 27.3%), Norway (11/47, 23.4%), and Sweden (13/50, 26.0%).

## Discussion

Our results showed that weight loss and improved co-morbidity were the two main reasons for having surgery among women in all five countries, except for in Germany where pain reduction was ranked higher than weight loss. Participants in Sweden had the highest expectation on weight loss and expected to lose 94.3% of their excessive weight after surgery, compared to an expected EWL between 70.8 and 83.0% in the other countries. There were differences in the expectations of surgery vs. lifestyle as the main driver of weight loss and the expected impact of surgery was higher in Germany and the Netherlands compared to in Finland, Norway, and Sweden where participants expected lifestyle changes to have a large impact as well.

Overall, characteristics of participants in our study were comparable to those in previously published studies including patients undergoing bariatric surgery [31–33]. Although these studies included both men and women, participants were predominantly female. Despite being an international, multicenter study, characteristics of participants in the different countries were relatively similar with the exception of participants in Sweden having a lower BMI and reporting noticeable fewer co-morbidities such as type 2 diabetes and hypertension.

Different referral pathways prior to surgery in the participating countries may have influenced the selection of patients and contributed to the variations in prevalence of co-morbidities seen in the included countries. In Finland and Norway, where diabetes and hypertension were common, all patients had an evaluation by an endocrinologist before referral for surgical evaluation. Therefore, those admitted to surgery might have been the individuals most resistant to conventional weight loss and diabetes treatment, and with the most co-morbidities [34]. In Germany and the Netherlands, patients could be referred to surgical evaluation directly from the general practitioner, but thereafter underwent a psychiatric and endocrine evaluation and had a diet program prior to surgery. Unlike the other countries, most of the patients in Sweden were referred directly from the general practitioner independent of co-morbidity burden without endocrinological evaluation. At the Swedish recruitment site, there were also additional clinical studies specifically recruiting those with type 2 diabetes. Although nothing prevented patients from participating in multiple studies, participants with type 2 diabetes may have chosen not to participate in our study because they were already part of another study. The fact that we have no record of the number of patients invited, and therefore cannot compare patients that chose to participate with those choosing not to do so, is a limitation that should be acknowledged. However, given the different referral pathways in the included countries, it was not feasible to collect such information.

Our results showed that patients perceived many reasons as very important. When asked to rank only the top three reasons, however, weight loss and improved co-morbidities were the two main reasons for having surgery among women in all countries. This is in line with previous research where it has been well established that physical health and improved co-morbidities are important factors for patients to undergo bariatric surgery [11, 22–25, 35–38]. It should, however, be noted that the reasons that participants were asked to rank are a mix of causal and effect variables, for example, weight loss will affect co-morbidities that in turn may affect other variables. Therefore, patients may intuitively rank weight loss and improved co-morbidities high. Notably in our study is that improved co-morbidity was ranked as an important reason also among Swedish patients that reported lower levels of co-morbidities than the other countries. This shows that improved co-morbidity is an important reason also among those with less co-morbidities. Interestingly, in a follow-up of the Swedish sub-sample done 1-year post-surgery, women were most satisfied with their improved self-esteem [20].

Participants in our study expected to lose between 70 and 94% of their excess weight after surgery. Swedish participants had the highest expectations (94.3%) and Finnish participants had the lowest (70.8%). Expectations on absolute weight loss among participants in the different countries in our study are mirrored in participants reported body images, i.e., Stunkard silhouettes. The levels of expected %EWL are similar to those seen in other studies [9, 11, 35], or somewhat lower [10]. Contrary to finding by Heinberg et al. [10], participants with a lower BMI had higher expectations on weight loss in our study. Nevertheless, expectations are generally very high among all and likely do not match true levels of weight loss. As we are limited by the lack prospective follow-up of participants after surgery, we have not been able to make comparisons between expected and actual weight loss. The fact that we lack information on type of surgery that patients in our study underwent, also means that we cannot compare expected weight loss with what is considered a realistic weight loss after different procedures [10]. However, in the follow-up of the Swedish sub-sample conducted 1-year post-surgery, the mean percentage of excessive weight loss was high (86.9%), yet not fully reaching the expected excessive weight loss of 94.3% [20]. Further, Aelfers et al. [13] followed more than 600 patients during 2 years after bariatric surgery and showed percentages of EWL of 66.8% and 69.4% at follow-up 12 and 24 months after surgery, with 63.3% of patients overestimating their expected weight loss. Similar levels of %EWL have been shown by others as well [31]. These levels of %EWL correspond to what patients in previous studies have deemed as an “acceptable” weight loss [9, 12].

Among participants in Germany and the Netherlands, a majority of patients expected weight loss to be almost exclusively due to surgery and not lifestyle changes. In Finland, Norway, and Sweden, lifestyle resulting weight loss was more often attributed to surgery and lifestyle changes combined. It should be noted that although the comprehensibility of the study questionnaire was evaluated in pilot study, the question on the impact of surgery vs. lifestyle changes on weight loss has not been validated more extensively and results should be interpreted with that in mind. Nevertheless, the different referral pathways prior to surgery, as discussed above, may be one explanation to the difference in expectations seen. The fact that many patients contribute 100% of the resulting weight loss to surgery is interesting as the prevailing view among physicians is that lifestyle changes are inevitable, even though a change in eating behavior is due to the surgical procedure, in order to achieve long-term weight loss results after surgery [39, 40]. However, although patients may be aware of the impact of lifestyle, they still have unrealistic expectations on surgery. For example, in a qualitative study comprising 18 adults accepted for bariatric surgery in England, Homer et al. [41] showed that although all participants acknowledged that changes in diet and physical activity were needed for long-term success of the surgery, some reported the unrealistic view that surgery would remove their own need to decide if to eat or not, while others recognized that personal control after surgery would still be needed.

The international aspect and multicenter design are strengths of this study. Nevertheless, it should be noted that the included countries have similarities both in terms of welfare status and culture, which limits the generalizability of our results. Another strength is the high completeness of data with only a small proportion of missing data. The customized and comprehensive questionnaire that was specifically designed for use in all five countries, and evaluated within each country, is also a strength. A limitation to the questionnaire may be that there was no time limit specified for when the expected weight loss should be reached, participants may therefore have interpreted the time interval differently. However, we believe that this would likely be random and not country specific.

A limitation of our study is that our results only can be generalized to women. Nevertheless, women are in majority among patients undergoing bariatric surgery [42]. Because only 50 participants from each country were going to be recruited in our study, the proportion of men would likely have been too small within each country to enable comparisons of men between the countries even if men would have been accepted into the study. The motivations of males seeking bariatric surgery have been studied by others [38], and in the comparison between women and men made by Fischer et al. [26], both ranked improved co-morbidities as the most important reason to seek surgery.

## Conclusion

Weight loss and improved co-morbidities were the main reasons for undergoing bariatric surgery in all five countries. Expectations on weight loss differed slightly between countries, but were in general very high. There was a difference in how much participants expected surgery, as opposed to lifestyle changes, to be the main driver of weight loss after surgery. It is important that patients understand the importance of lifestyle change and maintenance of a healthy lifestyle after surgery, in order to obtain a successful weight loss and avoid weight regain. While some patients are well informed, other may need additional counselling, since weight regain after bariatric surgery has been shown to be unexpected and patients have reported to be unprepared having unrealistic weight loss expectations [43, 44]. A future challenge will be to efficiently find the patients in need of additional support before, as well as after, surgery. To further address the effect of expectations on outcomes such as weight loss and weight maintenance after bariatric surgery, prospective studies with repeated and long-term follow-up of patients, including both women and men, that have undergone different types of bariatric surgical procedures are needed.

## Supplementary Information

Below is the link to the electronic supplementary material.

Supplementary file1 (DOCX 17 KB)
