# Supplementary material for: Women’s Reasons to Seek Bariatric Surgery and Their Expectations on the Surgery Outcome — a Multicenter Study from Five European Countries
Source: Obes Surg. 2022 Sep 23;32(11):3722–31. doi: 10.1007/s11695-022-06280-w (PMC9613564; doi:10.1007/s11695-022-06280-w)
Supplement: Supplementary file 1 — Supplementary file1 (DOCX 17 KB) [file 11695_2022_6280_MOESM1_ESM.docx]

| **Supplementary Table 1**. Number of participants ranking each reason to seek surgery as “very important”, i.e. as a 5 on the Likert scale, listed by order of ranking parameters. | | | | | | | | | | | | |
| --- | --- | --- | --- | --- | --- | --- | --- | --- | --- | --- | --- | --- |
| **Item to improve** | **All** | | **Finland** | | **Germany** | | **Norway** | | **Sweden** | | **the Netherlands** | |
|  | **n** | **(%)** | **n** | **(%)** | **n** | **(%)** | **n** | **(%)** | **n** | **(%)** | **n** | **(%)** |
| Weight loss | 200 | (80.0) | 39 | (78.0) | 41 | (82.0) | 42 | (84.0) | 38 | (76.9) | 40 | (80.0) |
| Increased life expectancy | 189 | (75.6) | 33 | (66.0) | 40 | (80.0) | 41 | (82.0) | 36 | (72.0) | 39 | (78.0) |
| Improved co-morbidity^1, 2^ | 164 | (66.4) | 38 | (77.6) | 40 | (80.0) | 29 | (60.4) | 33 | (66.0) | 24 | (48.0) |
| Improved physical activity^3^ | 145 | (58.5) | 31 | (63.3) | 41 | (82.0) | 27 | (54.0) | 36 | (73.5) | 10 | (20.0) |
| Improved self esteem^3^ | 131 | (52.8) | 25 | (51.0) | 30 | (60.0) | 28 | (57.1) | 31 | (62.0) | 17 | (34.0) |
| Improved mental health^4^ | 123 | (50.0) | 24 | (50.0) | 27 | (54.0) | 12 | (25.0) | 34 | (68.0) | 26 | (52.0) |
| Pain reduction^2^ | 123 | (49.8) | 25 | (52.1) | 36 | (72.0) | 20 | (40.8) | 25 | (50.0) | 17 | (34.0) |
| Reduced need for medication | 115 | (46.0) | 25 | (50.0) | 29 | (58.0) | 24 | (48.0) | 18 | (36.0) | 19 | (38.0) |
| Reduction in clothing size^2^ | 112 | (45.3) | 23 | (46.9) | 22 | (44.0) | 12 | (25.0) | 29 | (58.0) | 26 | (52.0) |
| Improved intimacy and partnership^3^ | 106 | (42.7) | 21 | (42.9) | 23 | (46.0) | 23 | (46.9) | 27 | (54.0) | 12 | (24.0) |
| Better work performance^4^ | 104 | (42.3) | 24 | (50.0) | 29 | (58.0) | 23 | (47.9) | 19 | (38.0) | 9 | (18.0) |
| Improved social life^2^ | 96 | (38.9) | 19 | (38.8) | 24 | (48.0) | 24 | (49.0) | 21 | (42.9) | 8 | (16.0) |
| Increased chance of employment^2^ | 71 | (28.7) | 13 | (26.5) | 20 | (40.0) | 12 | (25.0) | 17 | (34.0) | 9 | (18.0) |
| Improved fertility^2^ | 37 | (15.0) | 6 | (12.5) | 8 | (16.0) | 4 | (8.2) | 11 | (22.0) | 8 | (16.0) |
| ^1^Such as diabetes, hypertension, high blood lipids, sleep apnea, etc; ^2^n=247; ^3^n=248; ^4^n=246; ^5^n=106. | | | | | | | | | | | | |
